# Excavating and Understanding the Eccentric: Textual Analysis, Gender and Ageing in British cinema

**DOI:** 10.1093/geroni/igaf122.383

**Published:** 2025-12-31

**Authors:** Sian Barber, Gemma Carney

**Affiliations:** Queen’s University Belfast, Belfast, Northern Ireland, United Kingdom; Queen’s University Belfast, Belfast, Northern Ireland, United Kingdom

## Abstract

In this paper we use our combined expertise in textual film analysis and social and cultural gerontology to interrogate visualisations of age and gender in Hampstead (2017) and The Lady in the Van (2015). Building on existing work which analyses film as a narrative (Oró-Piqueras and Casado-Gual, 2020) we engage with cinematography, mise-en-scène and aesthetics, viewing film as a vivid and moving imaginary of old age. In particular, we use Barber’s (2015) expertise in British cinema to link the performances of Smith (The Lady in the Van) and Keaton (Hampstead) to incarnations of ageing women which are deeply rooted in British cinema. Both films allow us to locate the main female characters in the precise analytical frame of British cinema. Both provide a visceral and visual representation of older women’s precarious property ownership in two contrasting visions of London. For British cinema-goers Maggie Smith’s struggle with homelessness, mental health difficulties and status as a single, childless older woman evoke the harsh realities of London’s property market whilst drawing on British social realist cinema. By contrast, Keaton’s ‘American in London’ story of widowhood, ageing and ultimate salvation through the love of Irish man Brendan Gleeson draws heavily on heritage cinema aesthetics and familiar rom-com tropes. After attending the session participants will be able to see that a filmic gaze offers a powerful means of de-constructing homogenization of old age well documented by scholars of the ‘silvering screen’ (Chivers, 2011).

